# A Time-Series Water Level Forecasting Model Based on Imputation and Variable Selection Method

**DOI:** 10.1155/2017/8734214

**Published:** 2017-11-09

**Authors:** Jun-He Yang, Ching-Hsue Cheng, Chia-Pan Chan

**Affiliations:** Department of Information Management, National Yunlin University of Science and Technology, Yunlin, Taiwan

## Abstract

Reservoirs are important for households and impact the national economy. This paper proposed a time-series forecasting model based on estimating a missing value followed by variable selection to forecast the reservoir's water level. This study collected data from the Taiwan Shimen Reservoir as well as daily atmospheric data from 2008 to 2015. The two datasets are concatenated into an integrated dataset based on ordering of the data as a research dataset. The proposed time-series forecasting model summarily has three foci. First, this study uses five imputation methods to directly delete the missing value. Second, we identified the key variable via factor analysis and then deleted the unimportant variables sequentially via the variable selection method. Finally, the proposed model uses a Random Forest to build the forecasting model of the reservoir's water level. This was done to compare with the listing method under the forecasting error. These experimental results indicate that the Random Forest forecasting model when applied to variable selection with full variables has better forecasting performance than the listing model. In addition, this experiment shows that the proposed variable selection can help determine five forecast methods used here to improve the forecasting capability.

## 1. Introduction

Shimen Reservoir is located between Taoyuan City and Hsinchu County in Taiwan. The Shimen Reservoir offers irrigation, hydroelectricity, water supply, flood control, tourism, and so on. This reservoir is very important to the area and offers livelihood, agriculture, flood control, and economic development. Thus, the authorities should plan and manage water resources comprehensively via accurate forecasting.

Previous studies of reservoir water levels have identified three important problems:

1. There are few studies of reservoir water levels: related studies [1–4] in the hydrological field use machine learning methods to forecast water levels. They focused on water level forecasting of the flood stages in pumping stations, reservoirs, lakes, basins, and so on. Most of the water level forecasting of these flood stages collected the data about typhoons, specific climate, seasonal rainfall, or water levels.
2. Only a few variables have been used in reservoir water level forecasting. The literature shows only a few related studies of forecasting [5, 6]. These used water level as the dependent variable, and the independent variable only has rainfall, water level, and the time lag of the combined two variables. Thus, a few independent variables were selected. It is difficult to determine the key variable set in the reservoir water level.
3. No imputation method used in datasets of reservoir water level: previous studies of water level forecasting in hydrological fields have shown that the collected data are noninterruptible and long-term, but most of them did not explain how to deal with the missing values from human error or mechanical failure.

To improve these problems, this study collected data on Taiwan Shimen Reservoir and the corresponding information on daily atmospheric datasets. The two datasets were concatenated into single dataset based on the date. Next, this study imputed missing values and selected a better imputation method to further build forecast models. We then evaluated the variables based on different models.

This paper includes five sections: Section 2 is related work; Section 3 proposes research methodology and introduces the concepts, imputation methods, variable selection, and forecasting model; Section 4 verifies the proposed model and compares with the listing models.  Section 5 concludes.

## 2. Related Work

This section introduces a forecast method of machine learning, imputation techniques, and variable selection.

### 2.1. Machine Learning Forecast (Regression)

#### 2.1.1. RBF Network

Radial Basis Function Networks were proposed by Broomhead and Lowe in 1988 [7]. RBF is a simple supervised learning feed forward network that avoids iterative training processes and trains the data at one stage [8]. The RBF Network is a type of ANN for applications to solve problems of supervised learning, for example, regression, classification, and time-series prediction [9]. The RBF Network consists of three layers: input layer, hidden layer, and output layer. The input layer is the set of source nodes, the second layer is a hidden layer high dimension, and the output layer gives the response of the network to the activation patterns applied to the input layer [10]. The advantages of the RBF approach are the (partial) linearity in the parameters and the availability of fast and efficient training methods [11]. The use of radial basis functions results from a number of different concepts including function approximation, noisy interpolation, density estimation, and optimal classification theory [12].

#### 2.1.2. Kstar

The Kstar is an instance-based classifier that differs from other instance-based learners in that it uses an entropy-based distance function [13]. The Lazy Family Data Mining Classifiers supports incremental learning. It contains some classifiers such as Kstar, and it takes less time for training and more time for predicting [14]. It provides a consistent approach to handling symbolic attributes, real valued attributes, and missing values [15]. Kstar uses an entropy-based distance function for instance-based regression. The predicted class value of a test instance comes from values of training instances that are similar to the Kstar [16].

#### 2.1.3. KNN

The *k*-Nearest-Neighbor classifier offers a good classification accuracy rate for activity classification [17]. The kNN algorithm is based on the notion that similar instances have similar behavior and thus the new input instances are predicted according to the stored most similar neighboring instances [18].

### 2.2. Random Forest

A Random Forest can be applied for classification, regression, and unsupervised learning [19]. It is similar to the bagging method. Random Forest is an ensemble learning method. A decision tree represents the classifier. Random Forest gets *N* outputs through *N* decision trees. These are forecast by voting for all of the predicted results. It can solve the classification and regression problems. Random Forest is simple and easily parallelized [20].

### 2.3. Random Tree

Random Tree is an ensemble learning algorithm that generates many individual learners and employs a bagging idea to produce a random set of data in the construction of a decision tree [21]. Random Tree classifiers can deal with regression and classification problems. Random trees can be generated efficiently and can be combined into large sets of random trees. This generally leads to accurate models [22]. The Random Tree classifier takes the input feature vector and classifies it with every tree in the forest. It then outputs the class label that received the majority of the votes [23].

### 2.4. Imputation

The daily atmospheric data may have missing values due to human error or machine failure. Many previous studies have shown that the statistical bias occurred when the missing values were directly deleted. Thus, imputing data can significantly improve the quality of the dataset. Otherwise, biased results may cause poor performance in the ensuing constructs [24]. Single imputation methods have several advantages such as a wider scope than multiple imputation methods. Sometimes it is more important to find the missing values than to estimate the parameters [25]. The median of nearby point imputation methods uses nearby values for ordering and then selects the median to replace the missing value. The advantage of the median imputation method is that its replaced value is actually a real value in the data [26]. Series mean imputation methods replace the average of the variables directly. Regression imputation method uses simple linear regression to estimate missing values and replace them. The mean of the nearby point imputation methods is the mean of nearby values. The number of nearby values can be found by using a “span of nearby points” option [27]. The linear imputation is most readily applicable to continuous explanatory variables [28].

### 2.5. Variable Selection

The variable selection method mainly identifies the key variable that actually influences the forecasting target from several variables. It then deletes the unimportant variables to improve the model's efficiency. It can solve high dimensional and complex problems. Previous studies in several field have shown that variable selection can improve the forecasting efficiency of machine learning methods [29–32].

Variable selection is an important technique in data preprocessing. It removes irrelevant data and improves the accuracy and comprehensibility of the results [33]. Variable selection methods can be categorized into three classes: filter, wrapper, and embedded. Filter uses statistic methods to select variables. It has better generalization ability and lower computational demands. Wrapper methods use classifiers to identify the best subset components. The embedded method has a deeper interaction between variable selection and construction of the classifier [34].

Filter models utilize statistical techniques such as principal component analysis (PCA), factor analysis (FA), independent component analysis, and discriminate analysis in the investigation of other indirect performance measures. These are mostly based on distance and information measures [35].

PCA transforms a set of feature columns in the dataset into a projection of the feature space with lower dimensionality. FA is a generalization of PCA; the main difference between PCA and FA is that FA allows noise to have nonspherical shape while transforming the data. The main goal of both PCA and FA is to transform the coordinate system such that correlation between system variables is minimized [36].

There are several methods to decide how many factors have to be extracted. The most widely used method for determining the number of factors is using eigenvalues greater than one [10].

## 3. Proposed Model

**Figure 1:**
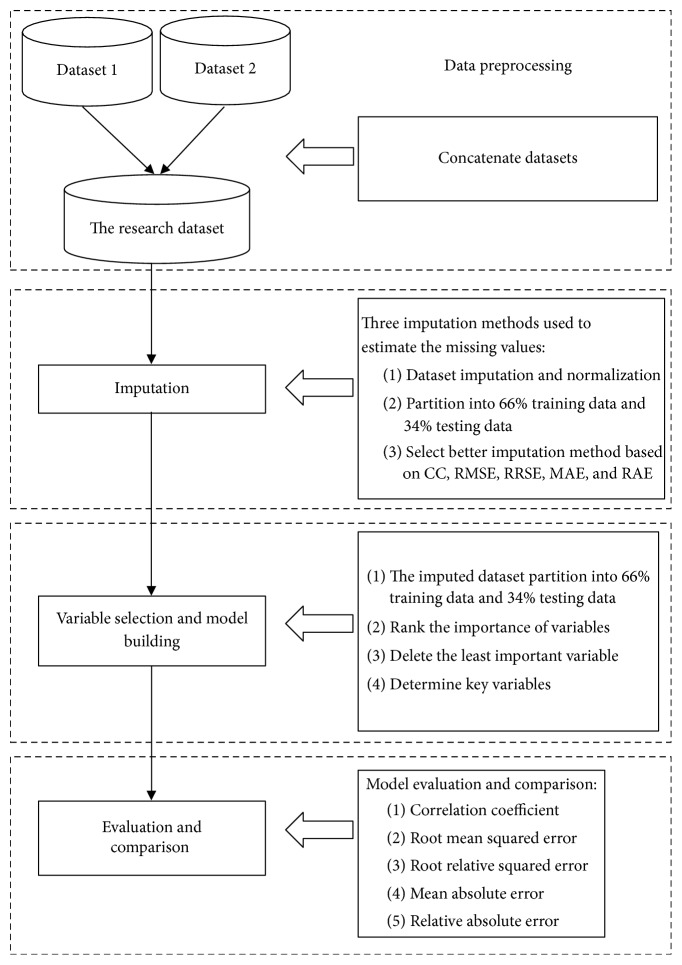
The procedure of proposed model.

Reservoirs are important domestically as well as in the national defense and for economic development. Thus, the reservoir water levels should be forecast over a long period of time, and water resources should be planned and managed comprehensively to reach great cost-effectiveness. This paper proposes a time-series forecasting model based on the imputation of missing values and variable selection. First, the proposed model used five imputation methods (i.e., median of nearby points, series mean, mean of nearby points, linear, and regression imputation). It then compares these findings with a delete strategy to estimate the missing values. Second, by identifying the key variable that influences the daily water levels, the proposed method ranked the importance of the atmospheric variables via factor analysis. It then sequentially removes the unimportant variables. Finally, the proposed model uses a Random Forest machine learning method to build a forecasting model of the reservoir water level to compare it to other methods. The proposed model could be partitioned into four parts: data preprocessing, imputation and feature selection, model building, and accuracy evaluation. The procedure is shown in Figure 1.

### 3.1. Computational Step

To understand the proposed model more easily, this section partitioned the proposed model into four steps.

**Step 1 (data preprocessing).**

The related water level and atmospheric data were collected from the reservoir administration website and the Taoyuan weather station. The two collected datasets are concatenated into an integrated dataset based on the date. There are nine independent variables and one dependent variable in the integrated dataset. The variables are defined in the integrated dataset and are listed in Table 1.

**Step 2 (imputation).**

After Step 1, we found some variables and records with missing values in the integrated dataset due to mechanical measurement failure or human operation error. Previous studies showed that deleting missing values directly will impact the results. To identify that one that better fits with the imputation method, this paper utilized five imputation methods to estimate the missing values and then compared it with no imputation method to directly delete the missing value. The five imputation methods were the median of the nearby points, series mean, mean of nearby points, linear imputation, and regression imputation. This study had six processed datasets after processing the missing values problem. The problem is then how to identify the imputation method that is a better fit to the integrated dataset. To determine this, the following steps were followed:

- In order to rescale all numeric values in the range [0, 1], this step normalized each variable value by dividing the maximal value for the five imputed datasets and then deleted the missing value dataset. All independent and dependent variables are positive values.
- The six normalized datasets are partitioned into 66% training datasets and 34% testing datasets. We also employed a 10-fold cross-validation approach to identify the imputation dataset that has best prediction performance.
- We utilized five forecast methods including Random Forest, RBF Network, Kstar, IBK (KNN), and Random Tree via five evaluation indices. These include correlation coefficient (CC), root mean squared error (RMSE), mean absolute error (MAE), relative absolute error (RAE), and root relative squared error (RRSE). We identified the normalized dataset with the smaller index value over five evaluation indices as well as the more fit imputation. Section 4 shows that the better imputation method is the mean of nearby points.

**Step 3 (variable selection and model building).**

Based on Step 2, this study will now determine the better imputation method (i.e., mean of nearby points). Then, the important problem is to determine the key variables that influence the reservoir water level. Therefore, this step utilized factor analysis to rank the importance of the variables for building the best forecast model. Based on the ordering of variable, we first deleted the least variable of importance. We then built the forecast model via a Random Forest, RBF Network, Kstar, KNN, and Random Tree. Next, we repeatedly delete the lower variable of importance to build the forecast model until the RMSE can no longer improve. After many iterative experiments, this study used five evaluation indices to determine the best forecast method. This step could be introduced step-by-step as follows:

- The imputed integrated datasets are partitioned into 66% training datasets and 34% testing datasets.
- Factor analysis ranked the importance of the variables.
- The variable ranking of factor analysis was used to iteratively delete the least important variable. The remaining variables were studied with Random Forest, RBF Network, Kstar, KNN, and Random Tree until the RMSE can no longer improve.
- Based on the previous Step 3, the key variables are found when the lowest RMSE is achieved.
- Concurrently, we used five evaluation indices (CC, RMSE, RRSE, MAE, and RAE) to determine which forecast method is a good forecasting model.

**Step 4 (evaluation and comparison).**

To verify the performance of the reservoir water level forecasting model, this step uses the superior imputed datasets with different variables selected to evaluate the proposed method. It then compares the results with the listing methods. This study used CC, RMSE, MAE, RAE, and RRSE to evaluate the forecast mode. The five criteria indices are listed as equations (1)–(5).

(1) $$\begin{array}{c} RMSE=\sqrt{\frac{1}{n}\overset{n}{\underset{i=1}{\sum }}{\left({\hat{y}}_{i}-y_{i}\right)}^{2}}, \end{array}$$

where *y*_*i*_ is the actual observation value of the data, ${\hat{y}}_{i}$ is the forecast value of the model, and *n* is the sample number.

*Correlation Coefficient (CC)*

(2) $$\begin{array}{c} CC=\frac{\sum_{i=1}^{N}\left(x_{i}-\overset{-}{x}\right)\cdot \left(y_{i}-\overset{-}{y}\right)}{\sqrt{\sum_{i=1}^{N}{\left(x_{i}-\overset{-}{x}\right)}^{2}}\cdot \sqrt{\sum_{i=1}^{N}{\left(y_{i}-\overset{-}{y}\right)}^{2}}}, \end{array}$$

where *x*_*i*_ and *y*_*i*_ are the observed and predicted values, respectively; $\overset{-}{x}$ and $\overset{-}{y}$ are the mean of the observed and predicted values.

*Root Relative Squared Error (RRSE)*

(3) $$\begin{array}{c} RRSE=\sqrt{\frac{\sum_{i=1}^{n}{\left(x_{i}-y_{i}\right)}^{2}}{\sum_{i=1}^{n}{\left(y_{i}-\overset{-}{y}\right)}^{2}}}, \end{array}$$

where *x*_*i*_ is the predicted value, *y*_*i*_ is the actual value, and $\overset{-}{y}$ is the mean of the actual value.

*Mean Absolute Error (MAE)*

(4) $$\begin{array}{c} MAE=\frac{1}{n}\times \overset{N}{\underset{i=1}{\sum }}\left|\;{\hat{y}}_{t}-y_{t}\;\right|, \end{array}$$

Here, *n* is the number of observation datasets, ${\hat{y}}_{t}$ is the forecast value at time *t*, and *y*_*t*_ is the actual value at time *t*.

*Relative Absolute Error (RAE)*

(5) $$\begin{array}{c} RAE=\frac{\sum_{i=1}^{n}\left|{\hat{y}}_{i}-y_{i}\right|}{\sum_{i=1}^{n}\left|{\overset{-}{y}}_{i}-y_{i}\right|}, \end{array}$$

where *y*_*i*_ is the actual observation value of the data, ${\overset{-}{y}}_{i}$ is the mean value of the $y_{i},{\hat{y}}_{i}$ is the forecast value of the model, and *n* is the sample number.

## 4. Experimental Results

This section verifies the performance of the proposed forecast model and compares the results with the listing methods. To determine which imputation method has the best performance for the collected dataset, this study collected daily atmospheric data from the monitoring station and the website of Water Resources Agency in Taiwan Shimen Reservoir. This work also compares the proposed model with the listing models with/without variable selection.

### 4.1. Experimental Data

The research dataset consisted of two historical datasets: one was collected form the website of Taiwan Water Resources Agency and the other was from Dasi monitoring station nearest to the Shimen Reservoir. The two datasets were collected from January 1, 2008, to October 31, 2015. The two collected data are concatenated into an integrated dataset based on the date. There are nine independent variables and one dependent variable in the integrated dataset that has 2,854 daily records. The study mainly forecasts the water level of the reservoir. The water level is the dependent variable. The independent variables are Reservoir_OUT, Reservoir_IN, Temperature, Rainfall, Pressure, Relative Humidity, Wind Speed, Direction, and Rainfall_Dasi, respectively. The detailed data types are shown as Table 2.

### 4.2. Forecast and Comparison

Based on the computational step in Section 3, this section will employ the practically collected dataset to illustrate the proposed model and compare it with the listing method. A detailed description is introduced in the following section.

1. To achieve better processing of the missing values dataset, this study applies series mean, regression, mean of nearby points, linear, or the median of nearby points' imputation methods to estimate missing values. It then directly deletes missing values to determine which method has the best performance. After normalizing the six processed missing values datasets, we used two approaches to estimate the datasets: percentage spilt (dataset partition into 66% training data and 34% testing data) and 10-fold cross-validation. The two approaches employ Random Forest, RBF Network, Kstar, KNN, and Random Tree to forecast water levels for evaluating the six processed missing values methods under five evaluation indices: CC, RMSE, MAE, RAE, and RRSE. The results are shown in Tables 3 and 4, and we can see that the mean of the nearby points' method wins versus other methods in CC, MAE, RAE, RRSE, and RMSE. Therefore, the mean of the nearby points' imputation method better estimates the Shimen Reservoir water level.
2. Variable selection and model building uses the results from above. The best imputation method is the mean of nearby points. Therefore, this study will use the imputed dataset of the mean of nearby points to select the variable and build the model. For variable selection, this study utilizes factor analysis to rank the importance of independent variables for an improved imputed dataset.  Table 5 indicates that the ordering of important variables is Reservoir_IN, Temperature, Reservoir_OUT, Pressure, Rainfall, Rainfall_Dasi, Relative Humidity, Wind Speed, and Direction.
3. Next, we determine the key variables and build a forecast model. This study utilizes the ordering of important variables and iteratively deletes the least important variable to iteratively implement the proposed forecasting model when the minimal RMSE is reached. First, all independent variables are used to build the water level forecasting model. Second, the least important variables are removed one-by-one. The remaining variables serve as a forecast model until the minimal RMSE is reached. After these iterative experiments, the optimal forecast model is achieved when the Wind Speed and Direction are deleted. The key remaining variables are Reservoir_IN, Temperature, Reservoir_OUT, Pressure, Rainfall, Rainfall_Dasi, and Relative Humidity. As a rule of thumb, we recommend interpreting only factor loadings with an absolute value greater than 0.5, which explains around 25% of the variance [37]. We can see that the loadings of the two deleted variables are smaller than 0.5 as seen in Table 5. Finally, this study employs the Random Forest forecast method based on variable selection and full variables to build a forecast model for water level forecasting in Shimen Reservoir, respectively.
4. Model comparison: this study compares the proposed forecast model (using Random Forest) with the Random Forest, RBF Network, Kstar, IBK (KNN), and Random Tree forecast models (Tables 6 and 7). Tables 6 and 7 show that before variable selection (full variables), the Random Forest forecast model has the best forecast performance in CC, RMSE, MAE, RAE, and RRSE indices. After variable selection, all five forecast models have improved forecast performance under CC, RMSE, MAE, RAE, and RRSE. Therefore, the results show that variable selection could improve the forecasting performance. The Random Forest model as applied to variable selection with full variables is better than the listing model.

### 4.3. Findings

After variable selection and model building, some key findings can be highlighted:

1. Imputation: after the two collected datasets were concatenated into an integrated dataset, there are missing values in the integrated dataset due to human error or mechanical failure. Tables 3 and 4 show the integrated dataset that uses the median of nearby points, series mean, mean of nearby points, linear, regression imputation, and the delete strategy to evaluate their accuracy via five machine learning forecast models. The results show that the integrated dataset that uses the mean of the nearby points' imputation method has better forecasting performance.
2. Variable selection: this study uses factor analysis to rank the ordering of variables and then sequentially deletes the least important variables until the forecasting performance no longer improves. Tables 6 and 7 show that the variable selection could significantly improve the performance of all five forecasting models. After iterative experiments and variable selection, the key remaining variables are Reservoir_IN, Temperature, Reservoir_OUT, Pressure, Rainfall, Rainfall_Dasi, and Relative Humidity.
3. Forecasting model: this study proposed a time-series forecasting model based on estimating missing values and variable selection to forecast the water level in the reservoir. These experimental results indicate that the Random Forest forecasting model when applied to variable selection with full variables has better forecasting performance than the listing models in the five evaluation indices. The proposed time-series forecasting model is feasible for forecasting water levels in Shimen Reservoir.

## 5. Conclusion

This study proposed a time-series forecasting model for water level forecasting in Taiwan's Shimen Reservoir. The experiments showed that the mean of nearby points' imputation method has the best performance. These experimental results indicate that the Random Forest forecasting model when applied to variable selection with full variables has better forecasting performance than the listing model. The key variables are Reservoir_IN, Temperature, Reservoir_OUT, Pressure, Rainfall, Rainfall_Dasi, and Relative Humidity. The proposed time-series forecasting model with/without variable selection has better forecasting performance than the listing models using the five evaluation indices. This shows that the proposed time-series forecasting model is feasible for forecasting water levels in Shimen Reservoir. Future work will address the following issues:

1. The reservoir's utility includes irrigation, domestic water supply, and electricity generation. The key variables identified here could improve forecasting in these fields.
2. We might apply the proposed time-series forecasting model based on imputation and variable selection to forecast the water level of lakes, salt water bodies, reservoirs, and so on.

In addition, this experiment shows that the proposed variable selection can help determine five forecast methods used here to improve the forecasting capability.

## Figures and Tables

**Table 1:** Description of variables in the research dataset.

Output	Shimen Reservoir daily discharge release
Input	Shimen Reservoir daily inflow discharge
Temperature	Daily temperature in Daxi, Taoyuan
Rainfall	The previous day Shimen Reservoir accumulated rainfall
Pressure	Daily barometric pressure in Daxi, Taoyuan
Relative Humidity	Daily relative humidity in Daxi, Taoyuan
Wind Speed	Daily wind speed in Daxi, Taoyuan
Direction	Daily wind direction in Daxi, Taoyuan
Rainfall_Dasi	Daily rainfall in Daxi, Taoyuan

**Table 2:** The partial collected data.

Date	Rainfall	Input	Output	Rainfall_Dasi	Temperature	Wind Speed	Direction	Pressure	Relative Humidity	Water level
2008/1/1	0.1	83.6		0	10.2	4.7	65	1001.5	56	244.09
2008/1/2	0.1	96.08	286.24	0	10.4	6.3	38	1000.7	59	243.93
2008/1/3	0	82.72	82.17	0	14.5	4.5	50	997.7	67	243.81
2008/1/4	0	133.32	262.22	0	15.3	3.4	40	996.5	82	243.78
2008/1/5	0	125.6	305.94	0	16	2.9	46	996.3	77	243.55
2008/1/6	0.3	98.74	192.33	0	16.7	1.2	170	995.4	83	243.32
2008/1/7	0	116.6	192.76	0	18.4	1.9	46	994.9	77	243.34
2008/1/8	0	93.12	109.73	0	19.9	1.3	311	992.9	78	243.33
2008/1/9	0	107.57	123.98	0	19.9	2.2	11	992.2	77	243.23
2008/1/10	0	65.15	276.74	0	19.6	1.6	357	991.6	80	243
2008/1/11	0	55.64	249.09	0	21.5	1.3	185	990.2	71	242.78
2008/1/12	0	91.67	191.81	0	19.8	4.2	37	992.1	75	242.74
2008/1/13	0.9	107.34	182.22	1.5	14.2	7.1	39	996.2	85	242.53
2008/1/14	5.2	80.09	146.62	1	12.7	7.1	36	997.7	85	242.49
2008/1/15	4	85.77	243.82	0	13.5	7.1	35	998.4	86	242.38

**Table 3:** The results of listing models with the five imputation methods under percentage spilt (dataset partition into 66% training data and 34% testing data) before variable selection.

	Methods	Index	RBF Network	Kstar	Random Forest	IBK	Random Tree
Before variable selection							
	Delete the rows with missing data	CC	0.085	0.546	0.728	0.288	0.534
		MAE	0.183	0.133	0.111	0.186	0.145
		RMSE	0.227	0.200	0.157	0.271	0.226
		RAE	0.998	0.727	0.607	1.015	0.789
		RRSE	0.997	0.879	0.690	1.193	0.995
	Serial mean	CC	0.052	0.557	0.739	0.198	0.563
		MAE	0.174	0.123	0.102	0.191	0.126
		RMSE	0.222	0.189	**0.151** ^*∗*^	0.283	0.202
		RAE	1.001	0.705	0.587	1.098	0.722
		RRSE	0.999	0.850	0.679	1.276	0.908
	Linear	CC	0.054	0.565	0.734	0.200	0.512
		MAE	0.175	0.121	0.101	0.189	0.138
		RMSE	0.222	0.188	0.152	0.281	0.218
		RAE	1.000	0.690	0.575^*∗*^	1.082	0.787
		RRSE	0.999	0.844	0.684	1.264	0.980
	Near median	CC	0.054	0.571	0.737	0.227	0.559
		MAE	0.175	0.120	0.101	0.188	0.126
		RMSE	0.222	0.186	0.152	0.277	0.202
		RAE	1.000	0.689	0.577	1.074	0.719
		RRSE	0.999	0.838	0.681	1.244	0.907
	Near mean	CC	0.053	0.572	**0.740** ^*∗*^	0.232	0.512
		MAE	0.175	0.121	**0.101** ^*∗*^	0.186	0.132
		RMSE	0.222	0.186	**0.151** ^*∗*^	0.275	0.217
		RAE	1.000	0.690	**0.575** ^*∗*^	1.062	0.756
		RRSE	0.999	0.837	**0.678** ^*∗*^	1.235	0.975
	Regression	CC	0.052	0.564	0.739	0.200	0.509
		MAE	0.174	0.121	0.102	0.191	0.133
		RMSE	0.222	0.188	0.151	0.283	0.216
		RAE	1.001	0.695	0.586	1.096	0.762
		RRSE	0.999	0.845	0.680	1.275	0.974

*∗* denotes the best performance among 5 imputation methods.

**Table 4:** The results of listing models with the five imputation methods under 10-folds cross-validation before variable selection.

	Methods	Index	RBF Network	Kstar	Random Forest	IBK	Random Tree
Before variable selection	Delete the rows with missing data	CC	0.041	0.590	0.737	0.246	0.505
		MAE	0.184	0.126	0.109	0.195	0.143
		RMSE	0.227	0.188	0.154	0.281	0.225
		RAE	1.000	0.682	0.592	1.059	0.775
		RRSE	0.999	0.825	0.678	1.235	0.986
	Serial mean	CC	0.038	0.612	0.755	0.237	0.574
		MAE	0.171	0.113	0.098	0.181	0.125
		RMSE	0.217	0.175	**0.143** ^*∗*^	0.270	0.202
		RAE	1.001	0.660	0.575	1.058	0.731
		RRSE	0.999	0.802	0.658	1.241	0.929
	Linear	CC	0.042	0.615	0.753	0.243	0.551
		MAE	0.173	0.112	0.098	0.181	0.127
		RMSE	0.218	0.175	0.144	0.269	0.207
		RAE	1.000	0.649	0.568	1.057	0.736
		RRSE	0.999	0.800	0.660	1.233	0.948
	Near median	CC	0.043	0.614	0.752	0.251	0.535
		MAE	0.173	0.113	0.098	0.180	0.131
		RMSE	0.218	0.175	0.144	0.268	0.211
		RAE	1.000	0.653	0.568	1.041	0.757
		RRSE	0.999	0.801	0.661	1.227	0.967
	Near mean	CC	0.043	0.613	**0.756** ^*∗*^	0.250	0.558
		MAE	0.173	0.113	**0.098** ^*∗*^	0.179	0.125
		RMSE	0.218	0.175	0.144	0.268	0.205
		RAE	1.000	0.654	**0.565** ^*∗*^	1.039	0.725
		RRSE	0.999	0.802	**0.657** ^*∗*^	1.226	0.937
	Regression	CC	0.038	0.618	0.754	0.240	0.522
		MAE	0.171	0.112	0.098	0.181	0.133
		RMSE	0.217	0.174	**0.143** ^*∗*^	0.270	0.214
		RAE	1.001	0.653	0.574	1.055	0.778
		RRSE	0.999	0.798	0.659	1.239	0.983

*∗* denotes the best performance among 5 imputation methods.

**Table 5:** The results of variable selection.

	Factor		
	1	2	3
Input	.**983**	.072	.164
Output	.**918**	.037	.071
Rainfall	.**717**	.064	.503
Temperature	.092	.**965**	−.149
Pressure	−.254	−.**844**	−.182
Wind Speed	.096	−.**377**	.052
Direction	.041	.**351**	−.067
Rainfall_Dasi	.290	.068	.**693**
Relative Humidity	.025	−.196	.**413**

*Note.* Each factor's highest factor loading appears in bold.

**Table 6:** The results of compare forecasting models under percentage spilt (dataset partition into 66% training data and 34% testing data) after variable selection.

	Methods	Index	RBF Network	Kstar	Random Forest	IBK	Random Tree
After variable selection	Delete the rows with missing data	CC	0.033	0.638^a^	0.729^a^	0.251	0.545^a^
		MAE	0.182^a^	0.121^a^	0.111^a^	0.199	0.135^a^
		RMSE	0.229	0.176^a^	0.156^a^	0.287	0.212^a^
		RAE	0.992^a^	0.657^a^	0.602^a^	1.083	0.736^a^
		RRSE	1.007	0.775^a^	0.688^a^	1.262	0.935^a^
	Series mean	CC	0.107^a^	0.661^a^	0.739^a^	0.242^a^	0.551
		MAE	0.172^a^	0.107^a^	0.101^a^	0.179^a^	0.129
		RMSE	0.221^a^	0.167^a^	0.151^a^	0.268^a^	0.205
		RAE	0.988^a^	0.615^a^	0.579^a^	1.027^a^	0.740
		RRSE	0.995^a^	0.753^a^	0.678^a^	1.208^a^	0.923
	Linear	CC	0.105^a^	0.666^a^	0.735^a^	0.258^a^	0.596^a^
		MAE	0.173^a^	0.106^a^	0.100^a^	0.175^a^	0.120^a^
		RMSE	0.221^a^	0.166^a^	0.151^a^	0.266^a^	0.196^a^
		RAE	0.987^a^	0.606^a^	0.572^a^	1.002^a^	0.683^a^
		RRSE	0.995^a^	0.748^a^	0.681^a^	1.198^a^	0.883^a^
	Median of nearby points	CC	0.106^a^	0.666^a^	0.740^a^	0.264^a^	0.553
		MAE	0.173^a^	0.107^a^	0.100^a^	0.177^a^	0.127
		RMSE	0.221^a^	0.166^a^	0.151^a^	0.266^a^	0.207
		RAE	0.987^a^	0.611^a^	0.571^a^	1.013^a^	0.723
		RRSE	0.995^a^	0.747^a^	0.677^a^	1.195^a^	0.932
	Mean of nearby points	CC	0.1059^a^	0.667^a^	**0.745** ^ab^	0.249^a^	0.540^a^
		MAE	0.173^a^	0.107^a^	**0.099** ^ab^	0.179^a^	0.129^a^
		RMSE	0.221^a^	0.166^a^	**0.149** ^ab^	0.268^a^	0.214^a^
		RAE	0.987^a^	0.611^a^	**0.565** ^ab^	1.025^a^	0.735^a^
		RRSE	0.995^a^	0.747^a^	**0.672** ^ab^	1.207^a^	0.962^a^
	Regression	CC	0.107^a^	0.663^a^	0.739^a^	0.242^a^	0.559^a^
		MAE	0.172^a^	0.106^a^	0.101^a^	0.179^a^	0.126^a^
		RMSE	0.221^a^	0.167^a^	0.151^a^	0.268^a^	0.200^a^
		RAE	0.987^a^	0.610^a^	0.581^a^	1.027^a^	0.723^a^
		RRSE	0.994^a^	0.752^a^	0.678^a^	1.207^a^	0.900^a^

a denotes after variable selection with enhancing performance; b denotes the best performance among 5 models after variable selection.

**Table 7:** The results of compare forecasting models under 10-folds cross-validation after variable selection.

	Methods	Index	RBF Network	Kstar	Random Forest	IBK	Random Tree
After variable selection	Delete the rows with missing data	CC	0.103^a^	0.665^a^	0.737^a^	0.233	0.529^a^
		MAE	0.181^a^	0.115^a^	0.108^a^	0.193^a^	0.143^a^
		RMSE	0.226^a^	0.171^a^	0.154^a^	0.282^a^	0.223^a^
		RAE	0.984^a^	0.627^a^	0.589^a^	1.047^a^	0.774^a^
		RRSE	0.994^a^	0.749^a^	0.677^a^	1.238	0.977^a^
	Series mean	CC	0.081^a^	0.688^a^	0.751^a^	0.295^a^	0.547
		MAE	0.169^a^	0.103^a^	0.098^a^	0.170^a^	0.131
		RMSE	0.217^a^	0.158^a^	0.144	0.260^a^	0.209
		RAE	0.988^a^	0.600^a^	0.571^a^	0.990^a^	0.767
		RRSE	0.996^a^	0.727^a^	0.661	1.193^a^	0.960
	Linear	CC	0.081^a^	0.692^a^	0.750	0.286^a^	0.551^a^
		MAE	0.171^a^	0.102^a^	0.098^a^	0.169^a^	0.128
		RMSE	0.218^a^	0.158^a^	0.145	0.261^a^	0.207^a^
		RAE	0.988^a^	0.590^a^	0.566^a^	0.981^a^	0.740
		RRSE	0.996^a^	0.723^a^	0.662	1.196^a^	0.948^a^
	Median of nearby points	CC	0.083^a^	0.692^a^	0.752^a^	0.305^a^	0.555^a^
		MAE	0.171^a^	0.102^a^	**0.097** ^ab^	0.169^a^	0.126^a^
		RMSE	0.218^a^	0.158^a^	0.144^a^	0.259^a^	0.208^a^
		RAE	0.987^a^	0.593^a^	**0.563** ^ab^	0.980^a^	0.732^a^
		RRSE	0.996^a^	0.722^a^	0.660^a^	1.186^a^	0.951^a^
	Mean of nearby points	CC	0.082^a^	0.694^a^	**0.753** ^b^	0.276^a^	0.537
		MAE	0.171^a^	0.102^a^	**0.097** ^ab^	0.171^a^	0.129
		RMSE	0.218^a^	0.157^a^	**0.144** ^ab^	0.263^a^	0.210
		RAE	0.988^a^	0.593^a^	0.564^a^	0.993^a^	0.747
		RRSE	0.996^a^	0.721^a^	**0.659** ^b^	1.204^a^	0.960
	Regression	CC	0.081^a^	0.690^a^	**0.753** ^b^	0.295^a^	0.572
		MAE	0.169^a^	0.102^a^	0.098^a^	0.169^a^	0.126
		RMSE	0.217^a^	0.158^a^	**0.144** ^ab^	0.259^a^	0.204
		RAE	0.988^a^	0.595^a^	0.571^a^	0.989^a^	0.735
		RRSE	0.996^a^	0.725^a^	**0.659** ^ab^	1.193^a^	0.938

a denotes after variable selection with enhancing performance; b denotes the best performance among 5 models after variable selection.
